# Analysis of invasive group A streptococcal puerperal sepsis in Calgary, Alberta: clinical consequences and policy implications

**DOI:** 10.1017/ice.2024.154

**Published:** 2024-09

**Authors:** Amro Qaddoura, Megan McQuiston, Gregory Tyrrell, Matthew Croxen, Vincent Li, Rhonda Demarco, Suzanne Pinfield, Ruziyya Ramazanova, Karen Hope, Edith-Rose Cairns, Judy MacDonald, Jia Hu, Oscar Larios, Joseph Kim, Bayan Missaghi, Joseph Vayalumkal, Irene Martin, Valerie Marsten, Jennifer Soucie, Robert Douglas Wilson, Colin Birch, John Conly

**Affiliations:** 1 Department of Medicine, University of Calgary and Alberta Health Services, Calgary, AB, Canada; 2 Women’s Health, Calgary Zone, Alberta Health Services, Calgary, AB, Canada; 3 Public Health Laboratories, Alberta Precision Laboratories, AHS-Edmonton, Edmonton, AB, Canada; 4 Department of Laboratory Medicine and Pathology, University of Alberta, Edmonton, AB, Canada; 5 Infection Prevention and Control, Alberta Health Services Calgary, AB, Canada; 6 Communicable Disease Control, Public Health, Calgary Zone, Alberta Health Services, Calgary, AB, Canada; 7 Department of Community Health Sciences, Cumming School of Medicine, University of Calgary, Calgary, AB, Canada; 8 Department of Pediatrics, University of Calgary and Calgary, Calgary, AB, Canada; 9 Li Ka Shing Institute of Virology, University of Alberta, Edmonton, AB, Canada; 10 Women and Children’s Health Research Institute, University of Alberta, Edmonton, AB, Canada; 11 National Microbiology Laboratory, Public Health Agency of Canada, Winnipeg, MB, Canada; 12 Department of Obstetrics and Gynecology, University of Calgary and Alberta Health Services, Calgary, AB, Canada

## Abstract

We analyzed invasive group A streptococcal puerperal sepsis cases in a large health zone in Alberta, Canada between 2013 and 2022. Of the 21 cases, 85.7% were adjudicated as hospital/delivery-acquired, with 2 clusters having identical isolates found through whole genome sequencing. We implemented policy interventions across Alberta aimed at preventing future infections.

## Introduction

Group A streptococci (GAS) cause infections ranging from uncomplicated pharyngitis to severe invasive GAS (iGAS) such as necrotizing fasciitis.^1^ Rates of iGAS infections have been rising in recent years.^2^ Puerperal sepsis (PS) is a potentially life-threatening infection in mothers following childbirth, with GAS being a common cause.^3^ Although rare in developed countries, it is a well-recognized postpartum complication with potentially devastating consequences including maternal death.^4^

GAS commonly colonize the throat or skin and are primarily transmitted by contact of mucosal membranes with infectious droplets.^1^ Mothers are vulnerable to peripartum GAS infections due to disrupted vaginal mucosal and/or perineal cutaneous barriers.^1,3,5^ Outbreaks of nosocomial postpartum iGAS infections continue to be reported.^6^ Case clustering of infections due to identical strains of GAS implies a common source in some instances, with health care workers (HCWs) who were asymptomatic carriers identified in several outbreaks.^3,5,6^ This clustering suggests that these infections are potentially preventable.^6,7^

With a perceived increase in hospital-associated PS cases, we sought to comprehensively analyze iGAS-PS cases within the Calgary Zone health region in Alberta, Canada using epidemiologic and molecular techniques. We incorporated items as outlined in the STROBE template (https://www.strobe-statement.org/checklists/) for the reporting of cohort studies.

## Methods

### Data sources

All cases occurred in the Calgary Zone between January 1, 2013 and December 31, 2022. In Alberta, iGAS infections are a notifiable disease to Public Health (PH).^8,9^ GAS isolates were identified by the microbiology laboratory and reported to PH. Clinical case data were collected by trained PH staff using a standardized notifiable disease reporting form with the data stored centrally at the Ministry of Health.^8^ Clinical data were then linked to laboratory data from Alberta Precision Laboratories for each patient. Live-birth data and population census were obtained from the Government of Alberta Open Data.^10^

### Case definitions

PS cases were defined as iGAS infections of the genital tract occurring from parturition to 6-weeks postpartum with two or more of the following: pelvic pain, fever, abnormal vaginal discharge and/or odor, or delay in uterine involution. Confirmed iGAS infections were defined as PS with identification of GAS from a normally sterile site (such as blood).^8^ Probable cases of iGAS infection were defined as severe disease using clinical and laboratory criteria (without another etiology/explanation) with isolation of GAS from a nonsterile site.^8^

Cases were further adjudicated as hospital/delivery-acquired or community-acquired using a 48-hour timeline from time of delivery to symptom onset. All cases were independently adjudicated by Infection Prevention and Control (IPC) personnel and PH officials, with further review by IPC physicians and Medical Officers of Health. Hand hygiene and personal protective equipment (PPE) use information was also collected from delivery records.

### Molecular analysis and isolate similarity

Isolates of GAS from patients and, where available, HCWs, were analyzed using molecular techniques, including whole genome sequencing (Supplemental files). Temporo-spatially related cases and clusters were also similarly analyzed.

### Research ethics and policy development

Cases of iGAS infection are notifiable under the Alberta Public Health Act, requiring investigation by PH. The data obtained from this study were used to facilitate the development of policy interventions with the aim to reduce the incidence of PS (Supplemental files).

## Results

### Descriptive epidemiologic data

**Figure 1.**
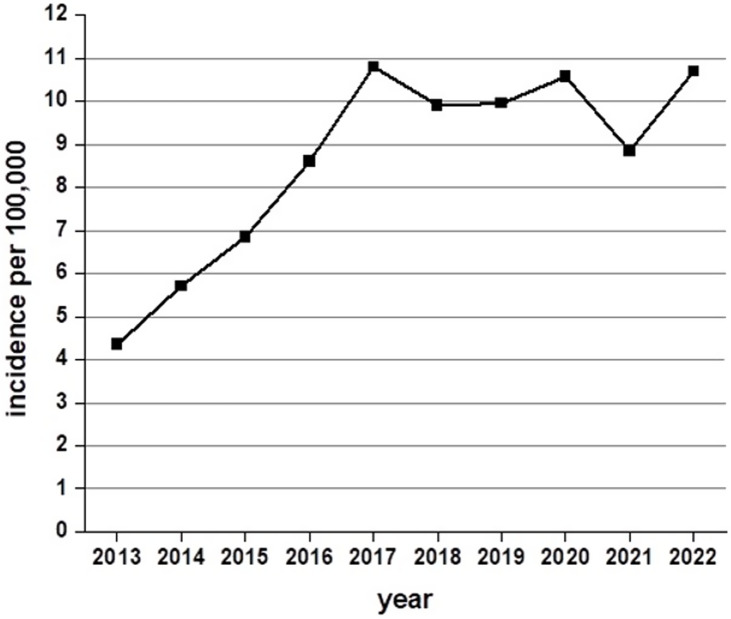
Rates of all iGAS infections (isolate based, counting one isolate per patient) in the province of Alberta (total population, adjusted by year, all age groups) 2013–2022 (ranging from 267 to 439 cases per year).

The rates of all iGAS infections in Alberta were increasing throughout the study period (Figure 1). Twenty-one iGAS-PS cases were identified between 2013 and 2022 in the Calgary Zone, representing a crude incidence of 1.35/10,000 live births. Of the 21 cases, there was one maternal death, nine adult intensive care unit (ICU) admissions, and six emergency hysterectomies (Supplemental Table).

Of the 21 cases, 18 (85.7%) were adjudicated as hospital/delivery-acquired, while three were community-acquired. Nineteen cases had iGAS isolates recovered, with 12 *emm* types identified (Table 1). The *emm* type could not be ascertained for two cases due to isolates not being submitted to the PH laboratory. Masks were not used during delivery for 14 (66.7%) cases, while information about PPE use was not available for six cases, and surgical mask use was confirmed for one case.

**Table 1.** iGAS-PS cases, year of occurrence, *emm* type, MLST, and presence or absence of streptococcal toxin genes

Case No.	year	Confirmed or Probable	HDA or CA	*emm* type	MLST	prophage encoded								chromosome encoded			
						*speA*	*speC*	*speH*	*speI*	*speK*	*speL*	*speM*	*ssa*	*speB*	speG	*speJ*	*smeZ*
1	2013	C	HDA	*emm*1.0	28	+								+	+	+	+
2	2013	C	HDA	*emm*1.0	28	+								+	+	+	+
3	2015	C	HDA	*emm*1.0	28	+								+	+	+	+
4	2022	C	CA	*emm*1.0	28	+								+	+	+	+
5	2017	C	HDA	*emm*4.0	39		+						+	+			+
6	2018	C	HDA	*emm*8.0	1190					+				+	+		+
7	2014	C	HDA	*emm*11.0	403		+				+	+		+	+		+
8	2014	C	HDA	*emm*11.0	403		+				+	+		+	+		+
9	2016	C	HDA	*emm*12.0	36		+	+	+					+	+		+
10	2017	P	HDA	*emm*12.8	36		+	+	+				+	+	+		+
11	2016	C	HDA	*emm*3.1	15					+			+	+	+		+
12	2018	C	HDA	*emm*28.0	458		+							+	+	+	+
13	2020	C	CA	*emm*28.0	458		+							+	+	+	+
14	2019	C	HDA	*emm*41.1	579		+				+	+		+	+		+
15	2014	C	HDA	*emm*75.0	None						+	+	+	+	+		+
16	2018	C	HDA	*emm*76.0	50			+	+					+	+	+	+
17	2016	C	HDA	*emm*82.0	334			+	+					+	+		+
18	2014	C	HDA	*emm*89.0	101		+							+	+		+
19	2017	P	CA	*emm*89.0	101		+							+	+		+
20	2014	C	HDA	NI	–												
21	2017	C	HDA	NI	–												
HCW	2013	–	–	*emm*1.0	28	+								+	+	+	+

C or P, confirmed or probable case of iGAS-PS; HCW, healthcare worker; HDA or CA, hospital/delivery-acquired or community-acquired; iGAS-PS, invasive Group A streptococcus puerperal sepsis; MLST, multilocus sequence typing; NI, no isolate.

### Cluster and molecular analysis

Four cases were identified as *emm*1 (cases 1–4, Table 1), with cases 1 and 2 occurring in spring 2013 at the same hospital. For cases 1 and 2, core single nucleotide variant (SNV) analysis showed the isolates to be identical to an isolate obtained from a pharyngeal swab of an unmasked HCW directly engaged in both deliveries. Cases 3 and 4 were not temporo-spatially linked to cases 1 and 2.

Cases 7 and 8 were typed as *emm*11 (Table 1). Both cases occurred in spring of 2014 at the same hospital and had the same healthcare team. The isolates matched through core SNV analysis. In addition, these cases were among those in which masking was not used. Case 18 was temporo-spatially linked to cases 7 and 8 (same month/same hospital/same healthcare team) but typed as *emm*89.

### Policy development

Policy changes addressing the rising rates of PS included multiple interventions, including masking, focused point-of-care risk assessment, and appropriate PPE for delivery personnel.

## Discussion

Our study identified 21 iGAS-PS cases between 2013 and 2022 which represented 1.35 cases/10,000 live births in the Calgary Zone health region and had unfortunate consequences including mortality, ICU admissions, and emergency hysterectomies. Most cases (85.7%) were adjudicated as healthcare/delivery-acquired. The clustering and identical match of cases 1 and 2 to an isolate from an unmasked delivering HCW supports that transmission occurred from the delivering HCW. Other results to highlight involved cases 7, 8, and 18. Although cases 7 and 8 had *emm*11 and were matched molecularly, the *emm*89 for case 18 did not match. However, it suggested a potential breakdown in IPC standards including non-masking that could have contributed to these cases.

Our data suggest that PS secondary to hospital/delivery-acquired iGAS may be preventable given the molecular matches, suggesting transmission from a common source. These data were used to inform a multidisciplinary task force to implement recommendations and practice policy changes across the entire province to prevent iGAS-PS (Supplemental files).

Adherence with these policies was overshadowed by the COVID-19 pandemic, which required masking and eye protection during all patient interactions. Although the incidence of PS declined dramatically after 2019, interpreting the reasons for this is difficult given the pandemic.

Our study has recognized limitations. The detection of all iGAS infections in a health region is challenging. Our surveillance methods likely underestimate the total number of infections. Early antimicrobial administration prior to culture collection can render cultures negative in otherwise positive cases. Furthermore, bacterial cultures are not always collected in cases of invasive postpartum infections since the most likely micro-organisms are well-defined with several acceptable empiric antimicrobial options.^1,4,7^ We were also conservative in our definition for confirmed iGAS infection as it had to be isolated from a normally sterile site. The limited availability of GAS isolates from HCWs further limited the analysis. These factors together contribute to underestimating the total number of cases. Our study was also retrospective and did not allow for additional collection of information. Despite these limitations, the available data including the molecular analysis provides compelling evidence for the hospital/delivery-associated transmission of iGAS.

In summary, PS secondary to hospital/delivery-acquired iGAS is a potentially preventable infection. Our study found that most cases were hospital/delivery-acquired and highlights the need for ongoing surveillance and provides strong rationale for developing policies to prevent iGAS-PS, with ongoing evaluation to assess whether policy interventions reduce the burden of iGAS-PS.

## Supporting information

Qaddoura et al. supplementary material Qaddoura et al. supplementary material

## Supplementary material

The supplementary material for this article can be found at https://doi.org/10.1017/ice.2024.154.
