# Supplementary material for: Analysis of invasive group A streptococcal puerperal sepsis in Calgary, Alberta: clinical consequences and policy implications
Source: Infect Control Hosp Epidemiol. 2024 Oct 10;45(9):1133–6. doi: 10.1017/ice.2024.154 (PMC11518661; doi:10.1017/ice.2024.154)
Supplement: Qaddoura et al. supplementary material [file S0899823X24001545sup001.docx]

Supplemental Files

**SUPPLEMENTAL METHODS**

Molecular Analysis

Isolates of GAS from patients and, where available, HCWs who were cultured as a part of an iGAS case investigation or if symptomatic, were analyzed using molecular techniques. As per the Alberta Public Health Disease Management Guidelines for iGAS, isolates of GAS were submitted to the Provincial Public Health Laboratory (Alberta Precision Laboratories (APL) for sequencing of the *emm* gene which encodes the cell surface M protein, using previously described techniques^1,2^. Whole genome sequencing (WGS) was performed by the National Microbiology Laboratory and the APL Public Health laboratory, details of which are provided below.

Data Analysis for Isolate Similarity

Both clinical and laboratory data were analyzed by assessing temporo-spatially related cases and clusters and comparing *emm* typing, multilocus sequence typing (MLST), single-nucleotide variant (SNV) analyses and virulence factor profiling to ascertain if there was a match between isolates found in any HCWs, other family members, and the affected patients.

Whole Genome Sequencing

Genome sequencing was performed by extracting DNA using the MagaZorb DNA Mini-Prep Kit (Promega). Briefly, colonies grown in Todd-Hewitt Broth were centrifuged at 6000 x g for 2 min and supernatant removed. Cells were washed in 12 mM Tris and then lysed in mutanolysin/hyaluronidase lysis solution (62 ml; 10ml 3000U/mL mutanolysin (Sigma), 2 ml 30 mg/mL hyaluronidase (Sigma), and 50 mL 10 mM Tris). Lysozyme (15 µL, 100 mg/mL; Sigma) was added and incubated for 1 hr at 37 °C with shaking at 700 rpm (Eppendorf ThermoMixer F1.5). Proteinase K solution (20 µL) and RNase A (20 µL, 20 mg/mL; Qiagen or Invitrogen) were added and the tubes were incubated at room temperature for 5 mins. ATL lysis buffer (200 µL) was added and tubes incubated for 2 hrs at 56 °C with shaking at 900 rpm (Eppendorf ThermoMixer F1.5). Extracts were centrifuged at 9000 x g for 2 mins and wash, binding, and elution steps were completed with the KingFisher mL Purification System (Thermo Scientific) with Qiagen Buffer EB. Extracted genomic DNA was prepared using a modified Illumina DNA Prep protocol (<https://www.medrxiv.org/content/10.1101/2022.02.07.22269672v1>) on an Eppendorf epMotion (Provincial Public Health Laboratory) or Illumina Nextera XT (National Microbiology Laboratory). Genomes were sequenced either by NextSeq 500/550 or a High Output Illumina MiniSeq.
Data Analysis for Similarity of Isolates

Raw sequence data quality was quality trimmed and filtered by fastp 0.23.4 (https://pubmed.ncbi.nlm.nih.gov/30423086/) and assessed for quality using seqkit v2.4.0 (https://pubmed.ncbi.nlm.nih.gov/27706213/) and multiqc v1.14 (<https://pubmed.ncbi.nlm.nih.gov/27312411/>) . Genomes were assembled with SPAdes v3.15.5 (https://pubmed.ncbi.nlm.nih.gov/22506599/) using the wrapper Shovill 1.1.0 (github.com/tseemann/shovill), with a minimum length cutoff of 300 bp. In silico emm-typing was performed using emm-typer (github.com/MDU-PHL/emmtyper), MLST performed with mlst v2.23.0 (github.com/tseemann/mlst), and virulence factor profiling using abricate 1.0.1 (github.com/tseemann/abricate) using the virulence factor database (<https://pubmed.ncbi.nlm.nih.gov/34850947/> ; database version: 2021-MAR-27). To check if any emm1.0 isolates were the M1UK variant (<https://pubmed.ncbi.nlm.nih.gov/31519541/> ), fastp-trimmed sequence data was mapped to the NCBI RefSeq emm1.0 genome MGAS5005 (NC_007297.2), and variants called using Snippy 4.6.0 (github.com/tseemann/snippy), followed by identification of the 27 M1UK-specific mutations described in Lanskey et al (2019). Genetic relatedness of the emm1.0 isolates was assessed by calling variants using the fastp-trimmed data mapped against the SC19-3814-A assembly, using Snippy 4.6.0. All isolates covered at least 99.7% of the reference genome. Single nucleotide variant (SNV) differences between each isolate were calculated using snp-dists 0.8.2 (github.com/tseemann/snp-dists) of the snippy-core SNV alignment. The GAS genomes (20 isolates) were uploaded to the NCBI SRA (<https://www.ncbi.nlm.nih.gov/sra>) as bioproject PRJNA1051113.

Research Ethics

Investigations of iGAS cases are conducted as part of the mandatory requirements for notifiable diseases under the Public Health Act in Alberta. This study also underwent screening by the ARECCI tool (A pRoject Ethics Community Consensus Initiative, <https://arecci.albertainnovates.ca/>) and was found to fit with quality improvement and thus not requiring ethics approval. This study was unfunded.

Policy Development

The data obtained from this study were used to facilitate the development of policy interventions with the aim to reduce the incidence of PS. Key stakeholders in policy development included Infection Prevention and Control (IPC), Obstetrics and Gynecology, Women’s Health, and Public Health.

**SUPPLEMENTAL RESULTS**
The Calgary Health Zone Region (Calgary and surrounding area) has a current population of 1.78 million people with a median age of 38 years (<http://www.ahw.gov.ab.ca/IHDA_Retrieval/>).

*emm*28

As *emm*28 has previously been reported to have a strong association with PS, we further explored the genomes of these two isolates (cases 12 and 13). While the cases were not temporo-spatially related (2018 and 2020), they did have the same ST (458) and the same toxin profiles (**Table 1**). In addition, both were *emm*28 subclade SC1B.

Policy Interventions

Our data demonstrated that the vast majority (85.7%) of cases were hospital/delivery-acquired as opposed to being community-acquired. We also demonstrated a clustering of cases through temporo-spatial relatedness and genome sequencing linking cases to healthcare providers. As iGAS-PS has devastating consequences for maternity patients post-partum, a multidisciplinary task force was established which led to recommendations to adhere to PPE use, including the use of delivery-based masking following the first cluster in 2013/14 and procedural practice policy changes for the prevention of iGAS-PS in maternity patients in the Calgary Zone. However, these local recommendations had variable adherence. A policy change was formally implemented in November 2019 through Infection Prevention and Control and was adopted provincially by the Maternal Newborn Child and Youth Strategic Care Network (MNCY SCN) in March 2020. This policy was in line with the American Academy of Pediatrics / American College of Obstetricians and Gynecologists perinatal care guidelines^3^.

The province-wide policy adoption occurred at a time when there was a crude rate of almost 1.4 iGAS-PS cases /10,000 live-births since 2013, and was comprised of several interventions, including education on the recognition of the signs and symptoms of PS, updating routine PPE practices at the time of delivery to include wearing a procedural mask if within two metres of the perineum during a vaginal delivery, self-assessments for fitness for work if attending deliveries, follow-up after iGAS exposure, and recommendations for managing patients and support people with symptoms of a respiratory tract infection. As well, it included a rationale behind each intervention, and a frequently asked questions section to help healthcare workers understand the importance of these interventions (included in Supplemental Files).

The next steps planned included assessing adherence with the introduced polices, but these efforts never occurred due to the Coronavirus Disease 2019 (COVID-19) pandemic that resulted in requirements for using surgical masks and eye protection during all patient interactions. The incidence of puerperal sepsis following the policy introduction in late 2019 was 0.6/10,000 live-births up to the end of 2022.

Supplement References

1. Centers for Disease Control and Prevention. Protocol for *emm* typing. Last reviewed: July 23, 2021. Available from: <https://www.cdc.gov/streplab/groupa-strep/emm-typing-protocol.html?CDC_AA_refVal=https%3A%2F%2Fwww.cdc.gov%2Fstreplab%2Fprotocol-emm-type.html> Accessed July 21 2024

2. Centers for Disease Control and Prevention. M Protein Gene (*emm*) Typing. Last reviewed: July 23, 2021. Available from: <https://www.cdc.gov/streplab/groupa-strep/emm-background.html>

Accessed July 21 2024

3. AAP Committee on Fetus and Newborn. (2017). *Guidelines for perinatal care*. American College of Obstetricians and Gynecologists Women’s Health Care Physicians. Chapter 13: Infection Control.

**Supplemental Table.** Additional information about the iGAS-PS cases.

| **Case No.** | **Year** | **C or P** | **HDA or CA** | **Culture Site*** | ***emm* Type** |
| --- | --- | --- | --- | --- | --- |
| 1 | 2013 | C | HDA | Blood | *emm*1.0 |
| 2 | 2013 | C | HDA | Blood | *emm*1.0 |
| 3 | 2015 | C | HDA | Blood | *emm*1.0 |
| 4 | 2022 | C | CA | Blood | *emm*1.0 |
| 5 | 2017 | C | HDA | Blood | *emm*4.0 |
| 6 | 2018 | C | HDA | Blood | *emm*8.0 |
| 7 | 2014 | C | HDA | Blood | *emm*11.0 |
| 8 | 2014 | C | HDA | Blood | *emm*11.0 |
| 9 | 2016 | C | HDA | Blood | *emm*12.0 |
| 10 | 2017 | P | HDA | Vaginal, urine | *emm*12.8 |
| 11 | 2016 | C | HDA | Blood | *emm*3.1 |
| 12 | 2018 | C | HDA | Blood | *emm*28.0 |
| 13 | 2020 | C | CA | Blood | *emm*28.0 |
| 14 | 2019 | C | HDA | Blood | *emm*41.1 |
| 15 | 2014 | C | HDA | Blood | *emm*75.0 |
| 16 | 2018 | C | HDA | Blood | *emm*76.0 |
| 17 | 2016 | C | HDA | Blood | *emm*82.0 |
| 18 | 2014 | C | HDA | Blood | *emm*89.0 |
| 19 | 2017 | P | CA | Urine | *emm*89.0 |
| 20 | 2014 | C | HDA | Muscle tissue | NI |
| 21 | 2017 | C | HDA | Blood | NI |
| HCW | 2013 | - | - | Pharynx | *emm*1.0 |

*in cases where it was isolated from both a sterile and non-sterile site, the sterile site is listed.

The age range was 18 to 41 years. There was one maternal death, nine adult intensive care unit (ICU) admissions with septic shock, 12 cases with sepsis with endometritis and/or necrotizing fasciitis/severe perineal cellulitis, and six emergency hysterectomies.

C or P = confirmed or probable case of iGAS-PS; HCW = healthcare worker; HDA or CA = hospital/delivery-acquired or community-acquired; iGAS-PS = invasive Group A streptococcus puerperal sepsis; NI = no isolate
